# Editorial: Adaptor Protein Regulation in Immune Signalling

**DOI:** 10.3389/fimmu.2020.00441

**Published:** 2020-03-13

**Authors:** Navin Kumar Verma, Thai Tran, Dermot Kelleher

**Affiliations:** ^1^Lee Kong Chian School of Medicine, Nanyang Technological University Singapore, Singapore, Singapore; ^2^Department of Physiology, Yong Loo Lin School of Medicine, National University of Singapore, Singapore, Singapore; ^3^Departments of Medicine and Biochemistry and Molecular Biology, University of British Columbia, Vancouver, BC, Canada

**Keywords:** innate and adaptive immunity, leukocyte motility, T cells, B cells, NK cells, mast cells, monocytes, CG-NAP

Adaptor proteins are essential cellular components that govern signaling cross-talks in time and space with precise specificity. They contain multiple protein-binding modules that bring cellular enzymes and effector molecules into close proximity to their targets, controlling their activities. These proteins add another layer to the specificity of signaling by the type of protein-binding modules they engage in and their subcellular localization. Several adaptor proteins have been identified that coordinate signal transduction cascades in immune cells for their effector functions, including motility, activation, proliferation, and differentiation.

This Research Topic is a collection of work that aims to provide an overview of emerging roles of adaptor proteins in immune functions. We invited several immunologists and scientists to update the knowledge about adaptor proteins in immune signaling. A total of six original research papers and three insightful reviews in this collection provide meaningful insights toward the roles of adaptor proteins in the functioning of various immune cell types, including T cells, B cells, natural killer (NK) cells, mast cells, and monocytes.

The topic starts with a primary article by Böning et al. in which authors demonstrate a crucial role of the adhesion and degranulation-promoting adapter protein (ADAP) in NK cell priming, cytokine production, and cytotoxicity in an *in vivo* setting. Using an intracellular pathogen *Listeria monocytogenes*, they show that infection-primed NK cells lacking ADAP produce inefficient amounts of perforin and have impaired cytotoxic capacity. In another original article, Rudolph et al. demonstrate that T cell-specific conditional ADAP knockout mice display less severe experimental autoimmune encephalomyelitis (EAE). They propose that ADAP-expressing NK cells and myeloid cells might synergistically contribute to the observed mild EAE. These datasets expand the knowledge about roles of the cytosolic adapter protein ADAP in immune cell functions.

The review by Verma et al. summarizes the role of CG-NAP/kinase interactions in T cell homeostasis and functions. Due to its ability to dynamically and spatial-temporally interact with multiple kinases, CG-NAP appears central to the functional regulation of T cell activation, proliferation, differentiation, and migration. They suggest exploiting CG-NAP/kinase interactions as tunable therapeutic targets for T cell-mediated diseases.

In the next very informative review, Yablonski describes the biological and functional roles of the Grb2-related adaptor downstream of Shc (Gads) in regulating allergy and T cell-mediated immunity. She expounds that linker for activation of T cells (LAT), Gads, and Src homology 2 (SH2) domain-containing leukocyte phosphoprotein of 76 kDa (SLP-76) form heterotrimeric microclusters that mediate signal transduction *via* the T cell receptor (TCR) and the mast cell high-affinity IgE receptor FcεRI. The review sheds light on additional Gads-binding molecules, including co-stimulatory proteins CD28 and CD6, adaptor protein Shc, deubiquitinating enzymes USP8 and AMSH, the serine/threonine hematopoietic progenitor kinase 1 (HPK1) and the tyrosine kinase BCR-ABL.

Natural killer T (NKT) cells are a distinct subset of T cells sharing phenotypic and functional characteristics common to both conventional T cells and NK cells. They can recognize lipid antigens presented by the major histocompatibility complex (MHC) class I-like CD1d molecules (1). The review by Gerth and Mattner drives the reader into the intracellular processes mediated by adaptor proteins, in particular the adaptor protein SLP-76, in the unique biology of NKT cells, such as selection, differentiation, and activation.

The toll-like receptor (TLR) eight is a known endosomal sensor of degraded RNA in human phagocytes and is involved in the recognition of viruses, bacteria, and mitochondria (2, 3). Using a TLR8 antagonist in their original article, Moen et al. demonstrate an important role of TLR8 in human monocytes challenged with *Staphylococcus aureus, Streptococcus agalactiae, Streptococcus pneumonia, Pseudomonas aeruginosa*, and *Escherichia coli*. They propose a novel signaling model where TLRs rapidly recruit and modify the interleukin-1 receptor-associated kinase 1 (IRAK-1) pool in monocytes, which may also sequester the adaptor protein MyD88 and/or IRAK-4, attenuating the interferon regulatory factor 5 (IRF5)-dependent cytokine induction and TLR8/IRF5 signaling.

The next original article by Zhou et al. demonstrates a vital role of the mitochondrial serine/threonine kinase phosphatase and tensin homolog (PTEN)-induced putative kinase 1 (PINK1) in innate antiviral immunity. The authors highlight that PINK1 positively regulates the retinoic acid-inducible gene I (RIG-I) triggered antiviral immunity by preventing the degradation of TNF receptor-associated factor 3 (TRAF3) and reducing the inhibition of the cellular responses mediated *via* the yes-associated protein (YAP).

Studies indicate that large antigen-containing particles, such as vaccinia virus, bacteria, and multicellular parasites, induce T cell-dependent B cell high-affinity antibody responses (4). Such responses require the internalization of large particulate antigens after the recognition by the B cell receptor (BCR). Using high-throughput quantitative image analysis and a panel of small molecule inhibitors, Verstegen et al. show that human B cells require IgM-BCR signaling *via* PI3K to efficiently engulf large anti-IgM-coated particles. This signaling cascade involves the cytoplasmic adaptor protein NCK in addition to the co-receptor CD19. They further demonstrate that the IgM-BCR/NCK signaling facilitates the activation of Rho family GTP-binding protein RAC1 to promote actin cytoskeleton remodeling necessary for particle internalization. They propose the NCK/PI3K/RAC1 signaling axis as an attractive target for biological intervention to prevent undesired antibody response to large particulate antigens.

The Skp1/Cul1/F-box ubiquitin ligase, β transducin repeat-containing protein (β-TrCP), regulates a diverse range of intracellular signaling pathways (5, 6). In this collection, Liu et al. demonstrate how β-TrCP restricts signal transduction *via* the TNF receptor-associated factor 6/IκB kinase (TRAF6/IKK) upstream of IκBα signaling induced by bacterial lipopolysaccharide, which is implicated in the regulation of inflammatory signaling by TLRs.

In conclusion, the collection of original articles and reviews provide new and valuable insight about the complex roles of adaptor proteins in immune regulation and also illustrate the important roles that these molecules play in immune function. We hope that this collection would inspire future research employing advance molecular and genetic tools to further dissect the interplay between adaptor proteins and their interacting partners in immune cells. In the near future, we anticipate much progress in this area of research, a greater appreciation of adaptor protein regulation of immune cells and the emergence of adaptor proteins as potential new targets for therapy.

## Author Contributions

All authors listed have made a substantial, direct and intellectual contribution to the work, and approved it for publication.

### Conflict of Interest

The authors declare that the research was conducted in the absence of any commercial or financial relationships that could be construed as a potential conflict of interest.
